# Surgical bleeding in patients undergoing posterior lumbar inter-body fusion surgery: a randomized clinical trial evaluating the effect of two mechanical ventilation mode types

**DOI:** 10.1186/s40001-023-01080-z

**Published:** 2023-03-12

**Authors:** Mohammad Hajijafari, Mohammad Hossein Ziloochi, Manoochehr Salimian, Esmaeil Fakharian

**Affiliations:** 1 Department of Anesthesiology, School of Medicine, Kashan University of Medical Sciences (KAUMS), Kashan, IR Iran; 2 Department of Community Medicine, School of Medicine, Kashan University of Medical Sciences (KAUMS), Kashan, IR Iran; 3 Trauma Research Center (TRC), and Department of Neurosurgery, Kashan University of Medical Sciences (KAUMS), Kashan, IR Iran

**Keywords:** Pressure-controlled ventilation, Volume-controlled ventilation, Posterior lumbar inter-body fusion, Surgical blood loss

## Abstract

**Background:**

The purpose of the study was to compare the effect of using volume-controlled ventilation (VCV) versus pressure-controlled ventilation (PCV) on blood loss in patients undergoing posterior lumbar inter-body fusion (PLIF) surgery.

**Methods:**

In a randomized, single-blinded, parallel design, 78 patients, candidates for PLIF surgery, were randomly allocated into two groups of 39 to be mechanically ventilated using VCV or PCV mode. All the patients were operated in prone position by one surgeon. Amount of intraoperative surgical bleeding, transfusion requirement, surgeon satisfaction, hemodynamic parameters, heart rate, and blood pressure were measured as outcomes.

**Results:**

PCV group showed slightly better outcomes than VCV group in terms of mean blood loss (431 cc vs. 465 cc), transfusion requirement (0.40 vs. 0.43 unit), and surgeon satisfaction (82.1% vs. 74.4%); however, the differences were not statistically significant. Diastolic blood pressure 90 and 105 min after induction were significantly lower in PCV group (*P* = 0.043–0.019, respectively); however, blood pressure at other times, hemoglobin levels, and mean heart rate were similar in two groups.

**Conclusions:**

In patients undergoing posterior lumbar inter-body fusion surgery, mode of ventilation cannot make significant difference in terms of blood loss; however, some minor benefits in outcomes may lead to the selection of PCV rather than VCV. More studies with larger sample size, and investigating more factors may be needed.

## Introduction

Demand for lumbar spine fusion, a common surgical procedure used for the treatment of many spinal conditions, has been increased in recent decades [1]. Although several surgical approaches exist for the inter-body fusion, posterior lumbar inter-body fusion (PLIF) is generally used due to its effective access and desirability of the surgical field [2]. PLIF surgery may improve physical activity and satisfaction of patients with chronic back pain [3]. However; surgical bleeding as a main concern in spine surgery has put lumbar spine fusion among the top 10 surgical procedures that necessitate blood transfusion [1, 4–9].

Reduction of surgical blood loss is crucial to maintain hemodynamic stability and the desirability of the surgical field. Meanwhile, convenience of the surgeon is associated with reduction of intraoperative blood loss due to reduced operation time [10]. Many factors contribute to the amount of intraoperative blood loss including sex, body mass index (BMI), the severity of deformity, surgical approach, number of levels, and anesthetic factors, such as the mode of mechanical ventilation [7, 11, 12].

Volume-controlled ventilation (VCV), which is known as the classic ventilation mode, has been used for decades with a constant flow to deliver a target tidal volume and thus ensure satisfactory minute ventilation; however, using VCV mode may result in high airway pressure levels when chest compliance decreased, such as in obese patients, and lead to ventilation induced lung injury [13, 14]. Limited inspiratory pressure in pressure-controlled ventilation (PCV), an alternative mode of mechanical ventilation, may reduce the risk of barotrauma and volutrauma. It also ensures that collapsed alveoli open up by extending the inspiratory time using sufficient positive end-expiratory pressure (PEEP) levels [15].

Although there are no remarkable differences between the two ventilation modes, some researchers have carefully recommended changing the ventilation settings from VCV to PCV in some situations [16, 17].

Surgical bleeding in prone position has been known to be associated with increase in intra-abdominal pressure (IAP), peak inspiratory pressure (PIP), and inferior vena cava (IVC) compression. Therefore, reduction of IAP, PIP, and IVC have been suggested to control intraoperative surgical blood loss. Some authors argued that PCV by providing lower PIP, compared with VCV, may be useful in control of surgical bleeding [18].

With regard to bleeding outcome, some studies have particularly shown that using PCV can reduce intraoperative blood loss [13, 19–22]; however, there are some controversies in this regard [23, 24].

Considering the above-mentioned controversies, we carried out the present study to investigate the effect of mechanical ventilation mode on the outcomes in patients undergoing posterior lumbar inter-body fusion (PLIF) in prone position. To reach this goal, we compared VCV and PCV in terms of surgical outcomes. Our primary outcome was the amount of intraoperative blood loss. Secondary outcomes were heart rate, blood pressure, transfusion requirement, and surgeon satisfaction.

## Methods

### Study design and population

This prospective, randomized, parallel and one-way blinded clinical trial, was conducted at Shahid Beheshti Hospital, a teaching hospital affiliated by Kashan University of Medical Sciences (KAUMS), Kashan, Iran; between 2017 and 2019.

**Fig. 1:**
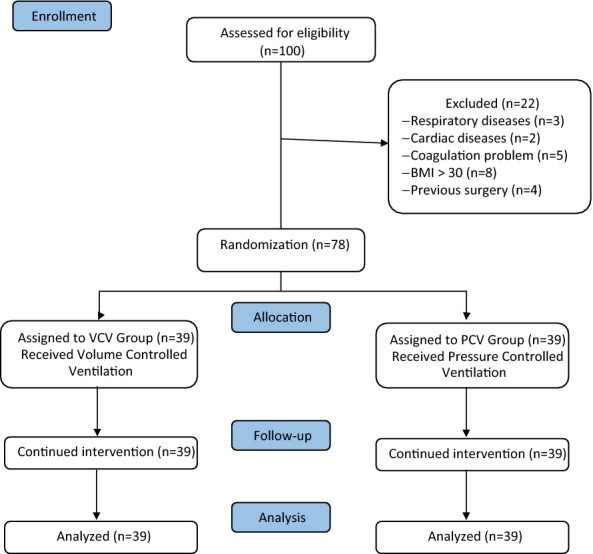
Consort flow diagram

To recruit the participants, 100 patients with ASA class 1 and 2, aged between 18 and 75 years, who were candidates for PLIF, were assessed. Among them, 22 patients were excluded. Exclusion criteria were: more than 6 planned surgical levels, respiratory diseases like asthma and COPD (*N* = 3), heart diseases such as heart failure, myocardial infarction, and valvar diseases (*N* = 2), coagulation diseases or anticoagulant consumption (*N* = 5), BMI > 30 (*N* = 8), and history of any previous lumbar or chest surgery (*N* = 4). After obtaining the written informed consent, the remained patients were randomly allocated into two groups of 39, namely VCV and PCV group (Fig. 1).

The permuted block randomization was used for the participants’ allocation (allocation ratio = 1:1). To do this, a computer-generated list and sealed envelopes were used. All the patients were operated in prone position by one surgeon and a single surgical team using the same method. Although the surgeon and involved nurses were aware of the study, they were blinded to the details of the study’s protocol.

### Ethics

This study was done from 2017 to 2019 at Shahid Beheshti Hospital, a teaching hospital affiliated by Kashan University of Medical Sciences (KAUMS). The study protocol was approved by the Ethics committee of KAUMS (Ethical code IR.KAUMS.REC.1395.50). All methods were carried out in accordance with relevant guidelines and regulations and with CONSORT recommendations.

### Anesthetic protocol

Attending anesthesiologist managed the anesthesia process. After establishing routine patient monitoring (heart rate, noninvasive blood pressure, pulse oximetry, and end tidal CO2), anesthesia was induced with an intravenous premedication of 2 μg/kg fentanyl, 0.03 mg/kg midazolam, 5 mg/kg sodium thiopental, and 0.5 mg/kg atracurium. Endotracheal tube with a suitable size, a high-volume, and a low-pressure cuff was inserted for the patients. Invasive monitoring of blood pressure through radial artery was applied for all patients after induction. Anesthesia was maintained with infusion of propofol 100–200 μg/kg/min as well as 30/70% of O2/N2O mixture to maintain MAP around 70 mmHg.

### Intervention

In PCV group, mechanical ventilation begun with PIP = 15 mmHg, RR = 12/min, I/E = ½, and PEEP = 3. Then the PIP was adjusted to achieve a tidal volume that was calculated as the ideal body weight (50 [female: 45.5] + 0.91 [height − 152.4]) × 7 ml. In addition, the respiratory rate was controlled, using the end-tidal carbon dioxide pressure (ETco2) ranged from 35 to 40 mmHg.

In VCV group, TV was set as 7 ml/IBW; however, RR, I/E, and PEEP were chosen as PCV group. Afterward, anesthesia was reversed with 0.04 mg/kg neostigmine and 0.02 mg/kg atropine.

### Outcome measures

Amount of intraoperative surgical bleeding, transfusion requirement, surgeon satisfaction, hemodynamic parameters, heart rate, blood pressure, and duration of surgery were measured. The amount of bleeding was estimated and registered based on the number of completely impregnated gauze pieces (15 cc for each 4 × 4 cm, and 50 cc for each 30 × 30 cm gauze piece), and the amount of blood in the suction bottle.

Transfusion threshold was based on MABL (maximum allowable blood loss) during surgery or Hb < 9 after that. Bleeding less than MABL was replaced with three times the Ringer's lactate volume. Further blood loss was replaced with transfusions of red blood cells.

Desirability of surgical field was recorded by the surgeon as good, moderate, or weak. Hemodynamic variables were recorded before the induction, after the intubation, after changing to prone position, every 15 min during the operation, at the end of surgery, after extubation, and before leaving the recovery unit. The surgeon and the staff who measured and recorded the hemodynamic variables were blinded to the mode of ventilation.

### Statistical analysis

SPSS-16 software was used to analyze the data. Normality of data was determined by Kolmogorov–Smirnov test. Statistical analysis was carried out using parametric (*t*-test and paired *t*-test) and non-parametric (Chi-square and Mann–Whitney) variance analysis. *P*-value < 0.05 was considered to be statistically significant.

## Results

During the study, 39 patients in each group of VCV and PCV were analyzed. The variables of sex, age, height, weight, and body mass index (BMI) were not significantly different between VCV and PCV groups; hence, two groups were comparable and similar in terms of demographic and anthropometric profiles (Table 1).

**Table1:** Demographic and anthropometric profiles

	VCV group (*n* = 39)	PCV group (*n* = 39)
Male	15(38.5%)	16(41%)
Female	24(61.5%)	23(59%)
Age (year)	42.41 ± 10.45	43.77 ± 11.38
Height(m)	1.65 ± .9	1.66 ± .1
Weight(kg)	76.11 ± 11.47	78.77 ± 19.71
BMI	27.92 ± 4.89	28.42 ± 7.10

In terms of number of surgical levels, although there were more patients with 4 levels in VCV group, two groups had no overall difference (Table 2).

**Table 2:** Number of surgical procedure levels in studied groups

Group	Number of levels					Sum	*P*
	2	3	4	5	6		
VCV	0	21	11	7	0	39	0.068
	0%	53.8%	28.2%	17.9%	0%	100%	
PCV	1	28	3	7	1	39	
	2.5%	70%%	7.5%	17.5%	2.5%	100%	
Sum	1	49	14	14	1	39	
	1.3%	17.7%	17.7%	62%	1.3%	100%	

Table 3 shows the amount of intraoperative surgical bleeding in the studied groups. Although PCV was associated with less bleeding (431.28 cc vs. 465.26 cc), the difference between two groups was not statistically significant (*p* = 0.67) (Table 3).

**Table 3:** Mean blood loss in the studied groups

Group	Mean	SD	*P* value
VCV	465.26	33.81	0.67
PCV	431.28	36.1	

Ten patients in VCV group and 9 cases in PCV group required transfusion of one or two units of packed-cell, with the median number of 2 units for both groups. The difference between number of packed-cell received by patients in two groups was not significant (*p* = 1) (Table 4).

**Table 4:** Packed-cell units received by patients

Group	0	1	2	Sum	*P* value
VCV	29(74.4%)	3(7.7%)	7(17.9%)	39(100%)	1
PCV	30(75%)	3(10%)	6(15%)	39(100%)	
Sum	59(74.7%)	6(8.9%)	13(16.5%)	78(100%)	

In terms of surgical field desirability, the surgeon evaluated 82.1% of PCV and 74.4% of VCV cases as “Good”; however, the difference between two groups was not significant (*p* = 0.58) (Table 5).

**Table 5:** Surgeon satisfaction

Sum	PCV group	VCV group	Surgeon satisfaction
Good	29(74.36%)	32(82.1%)	61(78.2%)
Moderate	9(23.8%)	7(17.9%)	16(20.5%)
Weak	1(2.56%)	0	1(1.3%)
Sum	39(100%)	39(100%)	78(100%)
*P* value	0/58		

The patients who needed antihypertensive agents during the surgery were just two cases in PCV group. Although this number in VCV group was twice, the statistical test did not indicate a significant difference between two groups (*p* = 0.22) (Table 6).

**Table 6:** Need for antihypertensive drugs during the surgery

Group	Need for blood pressure-lowering drugs	No need for blood pressure-lowering drugs	Sum
VCV	4(10.3%)	35(89.7%)	39(100%)
PCV	2(5.1%)	37(94.9%)	39(100%)
Sum	6(7.7%)	72(92.3%)	78(100%)
*P* value		0/22	

Like before surgery, measurement of mean hemoglobin in patients of two groups after the surgery showed no significant difference between two groups (Table 7).

**Table 7:** Mean hemoglobin of patients before and after surgery in the study groups

Time	Group	Mean	SD	*P* value
Before surgery	VCV	13.39	1.47	0.772
	PCV	13.48	1.41	
After surgery	VCV	11.92	1.43	0.775
	PCV	12.01	1.41	

There were no significant differences between measurements of mean heart rate in two groups. Values of systolic blood pressure were also similar in two groups. However, diastolic blood pressure 90 and 105 min. after induction were significantly lower in PCV group (*P* = 0.043–0.019, respectively) (Table 8).

**Table 8:** Diastolic blood pressure in studied groups throughout operative time

Time	VCV group		PCV group		*P* value
	Mean	SD	Mean	SD	
Before induction	87.03	13.61	83.62	11.63	0.238
After induction	74.23	14.16	71.44	12.64	0.361
After change of position (from supine to prone)	77.49	16.57	79.13	16.16	0.659
15 min	72.26	14.30	72.00	15.28	0.939
30 min	67.67	13.51	65.69	12.94	0.512
45 min	65.74	13.45	60.74	12.61	0.094
60 min	64.97	13.60	59.46	10.56	0.049
75 min	65.74	13.24	60.90	9.43	0.066
90 min	66.79	13.03	61.41	9.83	0.043
105 min	67.38	11.59	61.59	9.61	0.019
120 min	66.05	13.57	61.33	9.05	0.075
End of surgery	71.31	12.83	65.82	11.36	0.049
After repositioning (from prone to supine)	73.74	12.77	70.10	10.16	0.168
Extubation time	72.00	16.72	72.28	8.36	0.925
Recovery	74.87	10.78	72.03	8.42	0.198

## Discussion

This study was done to investigate the effect of mechanical ventilation mode on the outcomes in patients undergoing PLIF in prone position. As main outcomes, amount of intraoperative surgical bleeding, transfusion requirement, and surgeon satisfaction were compared in two groups of VCV and PCV. Hemodynamic parameters, heart rate, and blood pressure were also measured and compared in the two groups as other outcomes.

PCV group showed slightly better outcomes than VCV group in terms of mean blood loss (431 cc vs. 465 cc), transfusion requirement (0.40 vs. 0.43 unit), and satisfaction of the surgeon from surgical field (82.1% vs. 74.4%); however, the differences were not statistically significant.

Zhendan Peng et al. after a similar randomized controlled trial reported a similar result of less, but not significant blood loss in PCV group [13]. Also, Lauren K. Dunn et al. in a retrospective study on a large sample size found that neither mode of mechanical ventilation nor airway pressure was associated with surgical bleeding or transfusion requirements [23].

However, there are some studies in which PCV has been reported to be significantly associated with lesser intraoperative blood loss [19–21, 24]. Reduced intraoperative blood loss has been argued to be related to decreased intrathoracic pressure and improved venous drainage which may be in turn due to decreased peak inspiratory pressure (PIP) during PCV [18, 21, 22].

To explain the inconsistency of our results with studies that demonstrated significant lesser surgical bleeding during PCV, other factors influencing intraoperative blood loss should be considered. For example, age is an important factor that affects cardiovascular and coagulation status. The mean age of patients in our study was 42–44 years, while the patients in Kang et al. study aged more than 64 years in average. Moreover, all patients in our study were operated in prone position, whereas in some studies the patient’s position during the surgery had been different. Length of surgery and number of surgical levels are among other factors that were different in our study and can explain our different results regarding surgical blood loss. In our study, there were 11 patients with 4 fused levels in VCV group comparing 3 patients in PCV group. Although not significant and by chance, this may have a negative effect on blood loss.

In our study, diastolic blood pressures 90 and 105 min. after induction were significantly lower in PCV group (*P* = 0.043–0.019, respectively); however, blood pressure in other times, hemoglobin levels, and mean heart rate were similar in two groups. Jun Han et al. in a recently published systematic review and meta-analysis have presented that hemodynamics variables are not significantly related to mode of ventilation during spine surgery in prone position [24].

Some authors believe that because of hemodynamic stability and providing lower PIP, PCV is a better choice than VCV for patients undergoing lumbar spine fusion surgery [13, 21, 25, 26].

## Limitations

One of the limitations of the present study was the sample size. Facing the conditions of the COVID-19 pandemic in a general hospital caused severe restrictions on the admission of elective cases like spinal surgery.

Another limitation was about baseline data. As the admission of patients to the hospital took place the night before the surgery, we could not record the hemodynamic variables sooner than the day of surgery. Therefore, mean values of blood pressure and heart rate over several days prior to the surgery were not included in baseline data.

Blinding is another issue in randomized trials. In this study, the attending anesthesiologist personally allocated the patients in VCV or PCV group, thus he was aware of the mode of ventilation. Of course, estimating the amount of bleeding was done by a staff nurse who was blinded to the mode of ventilation.

In conclusion, this study suggests that mode of ventilation cannot make significant difference in terms of blood loss in patients undergoing posterior lumbar inter-body fusion surgery; however, some minor benefits in outcomes may lead to the selection of PCV rather than VCV. More studies with larger sample size, and investigating more factors may be needed.

## Data Availability

Data used in this study can be accessed from the corresponding author.

## References

[1] Ristagno G (2018). Red blood cell transfusion need for elective primary posterior lumbar fusion in a high-volume center for spine surgery. J Clin Med.

[2] Malhotra A (2016). Quantifying the amount of bleeding and associated changes in intra-abdominal pressure and mean airway pressure in patients undergoing lumbar fixation surgeries: a comparison of three positioning systems. Asian Spine J.

[3] Resnick DK (2005). Guidelines for the performance of fusion procedures for degenerative disease of the lumbar spine part 7: intractable low-back pain without stenosis or spondylolisthesis. J Neuro Spine.

[4] Yoo JS (2019). The use of tranexamic acid in spine surgery. Ann Transl Med.

[5] Norton RP (2013). Complications and intercenter variability of three-column resection osteotomies for spinal deformity surgery: a retrospective review of 423 patients. Evid Based Spine Care J.

[6] Horlocker TT (2001). The accuracy of coagulation tests during spinal fusion and instrumentation. Anesth Analg.

[7] Hu SS (2004). Blood loss in adult spinal surgery. Eur Spine J.

[8] Cole JW (2003). Aprotinin reduces blood loss during spinal surgery in children. Spine.

[9] Szpalski M, Gunzburg R, Sztern B (2004). An overview of blood-sparing techniques used in spine surgery during the perioperative period. Eur Spine J.

[10] Janatmakan F (2019). Comparing the effect of clonidine and dexmedetomidine on intraoperative bleeding in spine surgery. Anesthesiol Pain Med.

[11] Kelly MP (2014). Evaluation of complications and neurological deficits with three-column spine reconstructions for complex spinal deformity: a retrospective Scoli-RISK-1 study. Neurosurg Focus.

[12] Li Z-J (2013). Is tranexamic acid effective and safe in spinal surgery? A meta-analysis of randomized controlled trials. Eur Spine J.

[13] Peng Z (2021). The effects of volume-controlled ventilation versus pressure-controlled ventilation on hemodynamic and respiratory parameters in patients undergoing lumbar spine fusion surgery: a randomized controlled trial. Ann Palliat Med.

[14] Movassagi R (2017). Comparison of pressure vs. volume controlled ventilation on oxygenation parameters of obese patients undergoing laparoscopic cholecystectomy. Pakistan J Med Sci.

[15] Meininger D (2005). Positive end-expiratory pressure improves arterial oxygenation during prolonged pneumoperitoneum. Acta Anaesthesiol Scand.

[16] Jiang J (2016). Pressure-controlled versus volume-controlled ventilation for surgical patients: a systematic review and meta-analysis. J Cardiothorac Vasc Anesth.

[17] Al Shehri AM (2014). Right ventricular function during one-lung ventilation: effects of pressure-controlled and volume-controlled ventilation. J Cardiothorac Vasc Anesth.

[18] Kim HB (2021). Equal ratio ventilation reduces blood loss during posterior lumbar interbody fusion surgery. Spine.

[19] Kundra S (2021). Effects of ventilation mode type on intra-abdominal pressure and intra-operative blood loss in patients undergoing lumbar spine surgery: a randomised clinical study. Indian J Anaesth.

[20] El-Sayed AA, Arafa SK, El-Demerdash AM (2019). Pressure-controlled ventilation could decrease intraoperative blood loss and improve airway pressure measures during lumbar discectomy in the prone position: a comparison with volume-controlled ventilation mode. J Anaesthesiol Clin Pharmacol.

[21] Kang W-S (2016). Effect of mechanical ventilation mode type on intra-and postoperative blood loss in patients undergoing posterior lumbar interbody fusion surgery: a randomized controlled trial. Anesthesiology.

[22] LI, N. (2017). Comparison of effects of pressure controlled ventilation and volume controlled ventilation on perioperative blood loss of patients with posterior lumbar interbody fusion. J Med Res.

[23] Dunn LK (2020). Ventilator mode does not influence blood loss or transfusion requirements during major spine surgery: a retrospective study. Anesth Analg.

[24] Han J (2022). Volume-controlled ventilation versus pressure-controlled ventilation during spine surgery in the prone position: a meta-analysis. Ann Med Surg.

[25] Choi EM (2011). Comparison of volume-controlled and pressure-controlled ventilation in steep Trendelenburg position for robot-assisted laparoscopic radical prostatectomy. J Clin Anesth.

[26] Jo YY (2012). The effect of pressure-controlled ventilation on pulmonary mechanics in the prone position during posterior lumbar spine surgery: a comparison with volume-controlled ventilation. J Neurosurg Anesthesiol.

